# Clinical Features of Severe Malaria Associated with Death: A 13-Year Observational Study in The Gambia

**DOI:** 10.1371/journal.pone.0045645

**Published:** 2012-09-28

**Authors:** Muminatou Jallow, Climent Casals-Pascual, Hans Ackerman, Brigitte Walther, Michael Walther, Margaret Pinder, Fatou Sisay-Joof, Stanley Usen, Mariatou Jallow, Ismaela Abubakar, Rasaq Olaosebikan, Aminata Jobarteh, David J. Conway, Kalifa Bojang, Dominic Kwiatkowski

**Affiliations:** 1 Malaria Programme, MRC Laboratories, Banjul, The Gambia; 2 Wellcome Trust Centre for Human Genetics, Oxford, United Kingdom; 3 Laboratory of Malaria and Vector Research, National Institute of Allergy and Infectious Diseases, Rockville, Maryland, United States of America; 4 London School of Hygiene and Tropical Medicine, London, United Kingdom; 5 Royal Victoria Teaching Hospital, Banjul, The Gambia; University of Barcelona, Spain

## Abstract

**Background:**

Severe malaria (SM) is a major cause of death in sub-Saharan Africa. Identification of both specific and sensitive clinical features to predict death is needed to improve clinical management.

**Methods:**

A 13-year observational study was conducted from 1997 through 2009 of 2,901 children with SM enrolled at the Royal Victoria Teaching Hospital in The Gambia to identify sensitive and specific predictors of poor outcome in Gambian children with severe malaria between the ages 4 months to 14 years. We have measured the sensitivity and specificity of clinical features that predict death or development of neurological sequelae.

**Findings:**

Impaired consciousness (odds ratio {OR} 4.4 [95% confidence interval {CI}, 2.7–7.3]), respiratory distress (OR 2.4 [95%CI, 1.7–3.2]), hypoglycemia (OR 1.7 [95%CI, 1.2–2.3]), jaundice (OR 1.9 [95%CI, 1.2–2.9]) and renal failure (OR 11.1 [95%CI, 3.3–36.5]) were independently associated with death in children with SM. The clinical features that showed the highest sensitivity and specificity to predict death were respiratory distress (area under the curve 0.63 [95%CI, 0.60–0.65]) and impaired consciousness (AUC 0.61[95%CI, 0.59–0.63]), which were comparable to the ability of hyperlactatemia (blood lactate>5 mM) to predict death (AUC 0.64 [95%CI, 0.55–0.72]). A Blantyre coma score (BCS) of 2 or less had a sensitivity of 74% and specificity of 67% to predict death (AUC 0.70 [95% C.I. 0.68–0.72]), and sensitivity and specificity of 74% and 69%, respectively to predict development of neurological sequelae (AUC 0.72 [95% CI, 0.67–0.76]).The specificity of this BCS threshold to identify children at risk of dying improved in children less than 3 years of age (AUC 0.74, [95% C.I 0.71–0.76]).

**Conclusion:**

The BCS is a quantitative predictor of death. A BCS of 2 or less is the most sensitive and specific clinical feature to predict death or development of neurological sequelae in children with SM.

## Introduction

Worldwide, more than 2 billion people live in areas where *Plasmodium falciparum* is transmitted which causes approximately 600,000 deaths each year. The burden of malaria mortality is borne primarily by African children. [1].

The definition of severe malaria (SM) is based on clinical features associated with a poor outcome including prostration, impaired consciousness, convulsions, respiratory distress, anemia, jaundice and shock. This definition captures the majority of patients at risk of dying and identifies children who require hospitalization and parenteral treatment; however, in settings with severe resource limitations, a definition with a higher specificity may be required. In sub-Saharan Africa, the clinical syndromes associated with increased mortality are cerebral malaria (CM), repeated convulsions and metabolic complications such as hypoglycemia and lactic acidosis. [2] Neurological involvement in falciparum malaria is common and nearly a quarter of children who survive CM develop neurological sequelae. [3], [4] Children with impaired consciousness and respiratory distress are at the highest risk of death. [5] Survivors of repeated malaria infections gradually acquire protective anti-disease immunity and thus the clinical presentation of SM depends on the degree of immunity acquired and the transmission intensity, which varies across different sites in Africa. [6], [7].

In an effort to define the most sensitive and specific clinical and prognostic features of SM in The Gambia, we studied 2,901 children with SM who were admitted to the Royal Victoria Teaching Hospital from 1997 to 2009, a period during which the prevalence of parasitemia in the community was declining. We describe temporal changes in the age of presentation and mortality of SM in The Gambia over this thirteen-year period and identify clinical features of SM at the time of admission associated with death and neurological sequelae.

## Methods

### Study Population

The Gambia is a sub-Sahelian country located on the west coast of Africa with a single rainy season (June to October) followed by a long dry season. Malaria transmission is highly seasonal with the peak incidence of malaria cases in October and November.

The 2,901 study participants were children aged 4 months to 15 years admitted to the Royal Victoria Teaching Hospital (RVTH) from January 1997 to December 2009. The RVTH serves as a secondary health facility for the local inhabitants and as tertiary hospital for most of the country.

### Clinical Definitions

Children aged 4 months to 14 years were eligible for enrollment if they had a blood smear positive for asexual *P. falciparum* parasites and met one or more WHO criteria for SM: Coma (assessed by the Blantyre Coma Score [BCS]), [8] severe anemia (hemoglobin [Hb] <50 g/L or packed cell volume [PCV]<15), respiratory distress (defined by the presence of any of the following clinical features: costal indrawing, use of accessory muscles, nasal flaring, deep breathing), hypoglycemia (<2.2 mM), decompensated shock (systolic blood pressure less than 70 mmHg), repeated convulsions (>3 during a 24 h-period), acidosis (plasma bicarbonate <15 mmol/L) and hyperlactatemia (plasma lactate>5 mmol/L). [9] Supporting clinical features of SM included prostration (inability to sit unaided in a child older than 7 months), hyperparasitemia (parasite density ≥500,000 parasites/µl), jaundice (diagnosed by clinical inspection), renal failure (urine output of <12 ml/Kg over a 24 h-period was used to make the diagnosis but was not measured in most patients enrolled in the study) and hyperpyrexia (axillary temperature >40°C).

CM was defined as a BCS of 2 or less with any parasite density. The coma score was carried out at least 30 minutes after the last seizure and after correction of hypoglycemia, and at least 6 hours after treatment of seizures with anticonvulsants. Severe malarial anemia was defined as Hb 50 g/L or less with a parasite density greater than 2,500 parasites per µL. Patients were enrolled in the study if informed consent was given by the parent or guardian. Ethnicity was defined according to the father’s ethnic group and the main language spoken by the parents.

### Clinical Management

Clinical staff performed the admission history and physical examination and provided 24 hour care for all patients. Children with SM were treated with intramuscular (IM) quinine 20 mg/kg loading dose followed by 10 mg/kg every 12 hours. Children able to take oral medication were transitioned to oral quinine 10 mg/kg three times a day to complete 5 days of treatment. During the study period, 356 children with SM were enrolled in an open-label randomized trial and 170 received parenteral artesunate (2.4 mg/kg on admission, at 12 h and 24 h) and a full standard dose of oral artemether-lumefantrine before discharge (1·5/9 mg/kg twice daily for 3 days). [10] Children with suspected bacterial infection received intravenous chloramphenicol (100 mg/kg/day in four divided doses) for 7 days and penicillin G (200,000 IU/Kg/day, in four divided doses). Children with Hb <50 g/L received blood transfusions. Children with Hb 50–80 g/L were transfused at the discretion of the physician if they showed signs of respiratory distress, or for parasite densities greater than 500,000 /µL. Hypoglycemia was managed with 1 mg/kg of 50% glucose bolus and 4% glucose infusion. Convulsions were treated with diazepam (0.5 mg per kg rectally or 0.3 mg per kg intravenously) or intramuscular paraldehyde (0.1 ml per kilogram), while patients with two or more witnessed seizures were treated with phenobarbitone (10–15 mg/kg loading dose and 5 mg/kg daily). Fluids (4 percent glucose in 0.18 percent saline solution) were given intravenously to comatose patients. Lumbar puncture was performed to rule out meningitis unless clinically contraindicated.

### Laboratory Investigations

Hemoglobin was measured with a hematology analyzer (Coulter ® MD II, Coulter Corporation, USA), PCV was measured with heparinized capillary tubes (Hawksley Ltd., UK) and parasitemia was counted on Giemsa-stained thick and thin films. Thin films were obtained from 370 patients enrolled in the AQUAMAT study and these were read by project laboratory technicians. All thick blood smears were read independently by two laboratory technicians. If there was disagreement between their readings on parasite positivity or if the difference, slides were read by a third technician. Agreement was reached among the three technicians after the slides had been rechecked. Inter-observer variability was assessed for parasite counting as part of the standard procedures for microscopy slide readings. Blood gases and glucose were measured using a portable i-STAT machine (Abbott, US). Lactate measurements were carried out in capillary or venous blood using a portable lactate analyzer (ArkRay Lactate Pro, US). Project staff was trained to collect and record data in a standardized manner. In addition, all laboratory procedures were carried out by trained staff using standard operating procedures.

### Data Management and Statistical Analyses

The data were collected on standardized paper forms, double entered into a database and verified against the original. Univariate and multiple logistic regression models were fitted for all clinical variables to identify prognostic factors, using death and presence of neurological sequelae as outcome variables using a significance level of 0.05. Goodness-of-fit was assessed by the Hosmer-Lemeshow test (P>0.05). In the multivariate analyses, the independent variables were checked for interaction. Area under the receiver operating characteristic curves was used to compare the sensitivity and specificity of selected markers. The anthropometric measurement weight-for-age Z-score (WAZ) was calculated using the STATA macro “igrowup.ado”. [11].

### Ethical Approval

Joint Gambia Government/MRC Ethical Committee approval (protocol numbers 630 and 670) was for a study of host genetic factors that predispose to severe malaria in children. Our study reports all admissions of children with severe malaria to RVTH that took place within the context of this study. Written informed consent was obtained from the next of kin, carers or guardians on the behalf of the minors/children participants involved in the study.

## Results

### Demographic Characteristics of the Study Population

From January 1997 through December 2009, 2,901 Gambian children with SM were admitted to the pediatric ward of the Royal Victoria Teaching Hospital (RVTH) and enrolled in the study (Figure S1). Of these patients, 95% were referred from periurban community health centers within 33 km. SM was seasonal: the first cases presented during the summer months and the greatest number of cases was seen during October and November of each year. The years with the greatest number of enrollments were 1997 and 1998 with 506 and 578 enrollments, respectively. The fewest enrollments were seen in 2006 and 2009 with 52 and 55 enrollments, respectively. The overall trend was towards decreasing number of enrollments each year (Chi-square for trend, P<0.001). Severity also appeared to decline, as indicated by a decreasing proportion of cases meeting a criterion for one of the SM syndromes (BCS <3, respiratory distress, or Hb <50 g/L) over the thirteen-year study period (Chi-square for trend, P<0.001). This was accompanied by a decline in overall annual mortality rate that ranged from 22.4% in 1999 to 5.7% in 2007. The overall trend showed a decline in the mortality rate over the study period (Chi-square for trend, P  = 0.008).

The median age of the children was 45 months (IQR 27–71) and 48% were female (Table 1). The most common ethnicity was Mandinka (40%), followed by Jola (20%), Fula (15%), Wolof (14%), Serahuli (1%) and other ethnic groups (9%). Many of the children were malnourished, with 484 (32.9%) children meeting criteria for moderate to severe malnutrition (weight-for-age Z-score −2 to −3).

**Table 1:** Baseline Characteristics of the Study Population.

			Observations	Value
**Age in months, median (IQR)**		Median (IQR)	2,890	45 (27–71)
**Sex (female)**		%	2,901	47.7
**Mother’s Ethnicity**		Number (%)	2,492	
	Mandinka	Number (%)		996 (39.9)
	Wolof	Number (%)		366 (14.6)
	Fula	Number (%)		379 (15.2)
	Jola	Number (%)		506 (20.3)
	Serahuli	Number (%)		28 (1.12)
	Other	Number (%)		217 (8.71)
**Weight for age (WAZ)**		Z-score (SD)	2,045	−0.85 (2.93)
**Temperature (°C)**		Mean (SD)	2,872	38.1 (1.01)
**Heart Rate (beats per minute)**		Mean (SD)	2,892	135.3 (24.3)
**Systolic Blood Pressure (mmHg)**		Mean (SD)	461	97.8 (13.9)
**Respiratory Rate (breaths per minute)**		Median (IQR)	2,887	40 (34–52)
**Hemoglobin (g/dL)**		Mean (SD)	2,839	6.95 (2.48)
**Parasite density (parasites/µL)**		Geometric mean (95%CI)	2,828	33,049(30,692–35,588)

### Clinical Features of Severe Malaria

Of the 2,901 children enrolled in this study: 2,857 (99.7% [95%CI, 99.5–99.9]) presented with fever, 2,107 with prostration (94.1% [95%CI, 93.2–95.1]), 2,100 with impaired consciousness (BCS <5) (72.5% [95%CI, 70.8–74.1]), 1,116 with coma (BCS <2) ) (38.5% [95%CI, 36.7–40.3]), 720 with convulsions during admission (40.8% [95%CI, 38.5–43.1]), 1,166 with respiratory distress (40.1% [95%CI, 38.4–41.9]), and 682 with severe anemia (24% [95%CI, 22.4–25.5]). These common presenting features classified the patients into three overlapping clinical syndromes: 1,166 children had respiratory distress (40.1% [95%CI, 38.4–41.9]), 1,060 (36.5% [95%CI, 34.7–38.2]) had CM, and 659 (22.7% [95%CI, 21.1–24.2]) had severe anemia (Table 2 and Figure S2).

**Table 2:** Prevalence of clinical features in Gambian children admitted to hospital with severe malaria.

	Data available	Prevalence (%)	Mortality (%)	Relative Risk(95% C.I.)	*P* value
Prostration	2,237	2,107 (94.1)	306 (14.5)	3.7 (1.5–9.0)	0.007
Impaired consciousness	2,896	2,100 (72.5)	358 (17.12)	4.6 (3.2–6.7)	<0.001
Hyperlactatemia	467	257 (55.0)	39 (15.2)	1.3 (0.8–2.2)	0.18
Repeated convulsions	1,763	720 (40.8)	139 (19.4)	2.0 (1.5–2.5)	<0.001
Hepatomegaly	2,865	1,111 (38.7)	166 (14.9)	1.2 (1.0–1.4)	0.03
Coma	2,896	1,116 (38.5)	286 (25.7)	4.5 (3.6–5.6)	<0.001
Respiratory distress	2,270	701 (30.8)	196 (28.1)	3.4 (2.8–4.2)	<0.001
Severe anemia	2,839	682 (24.0)	59 (8.6)	0.5 (0.4–0.7)	<0.001
Acidosis	293	69 (23.5)	14 (20.2)	5.6 (2.4–12.9)	<0.001
Hypoglycemia	2,042	449 (21.9)	84 (18.8)	1.8 (1.4–2.3)	<0.001
Splenomegaly	2,865	482 (16.8)	43 (8.9)	0.6 (0.4–0.8)	<0.001
Jaundice	2,840	288 (10.1)	60 (20.8)	1.6 (1.2–2.1)	<0.001
Hyperparasitemia	2,840	115 (4.0)	25 (21.7)	1.6 (1.1–2.3)	0.007
Hypotensive Shock	461	14 (3.0)	3 (21.4)	2.8 (1.0–8.3)	0.05
Hyperpyrexia	2,872	56 (1.9)	10 (17.8)	1.3 (0.7–2.3)	0.33
Renal failure	2,729	17 (0.6)	9 (52.9)	5.1 (3.2–8.0)	<0.001

variables were defined as follows: Prostration, inability to sit (children aged >7 months); Impaired consciousness, BCS ≤4; Coma, BCS ≤2; Repeated convulsions, >3 in 24 h; Severe anemia (with any parasite density), Hb <5 g/dL or PCV <15; Respiratory distress, abnormal respiratory pattern (respiratory pattern values > or  = 3), grunting or use of accessory muscles of respiration, or abnormally deep (acidotic) breathing; Hypoglycemia ≤2.2 mM; Hyperlactatemia, plasma lactate >5 mM; Hyperpyrexia, temp>40°C; Hyperparasitemia, *P. falciparum* parasite density >500,000 /µl; Hypotensive shock, circulatory collapse with systolic blood pressure <50 mmHg; Hepatomegaly >2 cm below right costal margin; Splenomegaly >2 cm below left costal margin.

### Mortality of the Major Severe Malaria Syndromes

There were 387 deaths among 2,890 children admitted with SM for whom outcome data were available (13.3%, [95%CI, 12.1–14.6]). Children with CM had the highest case fatality rate ranging from 16% (15.9 [95%CI, 12–19]) for CM alone to 35% (35.5 [95%CI, 31.1–39.9]) when CM was accompanied by respiratory distress (Table 3 and Figure S2). Respiratory distress alone had a case fatality rate of 20.9% (95% CI, 18.6–23.3). The lowest case fatality rate was observed in children admitted with severe anemia as the only clinical feature (1.15%, [95%CI, 0 to 2.4]). The mortality of children with more than one coincident SM syndrome (25.2% [95%CI, 22.3–28.1]) was significantly greater than those with only one SM syndrome (10.3% [95%CI, 8.4–12.1], P<0.001).

**Table 3:** Clinical features in Gambian children with severe malaria independently associated with a fatal outcome.

	Number of observations	Odds ratio	[95%CI]	P value	Sensitivity	Specificity	AUC (95%CI)
Impaired consciousness	2,885	4.4	2.7–7.3	<0.001	92.5	30.6	0.61 (0.59–0.63)
Respiratory distress	2,890	2.4	1.7–3.2	<0.001	63.0	63.2	0.63 (0.60–0.65)
Hypoglycemia	2,037	1.7	1.2–2.3	0.001	33.7	79.7	0.56 (0.53–0.59)
Jaundice	2,830	1.9	1.2–2.9	0.002	15.7	90.6	0.53 (0.51–0.55)
Renal failure	2,725	11.1	3.3–36.5	<0.001	3.1	99.6	0.51 (0.50–0.52)

The multiple logistic regression analysis included 1,931 observations with complete data (5 degrees of freedom) χ^2^ = 180.4 (P<0.001); pseudo-R2 = 0.10; Goodness-of- fit, statistics: Hosmer-Lemeshow  = 4.47 (P = 0.61). AUC =  area under the curve.

A substantial number of children (N = 834) fit the WHO case definition of SM, but did not meet criteria for one of the three SM syndromes. This group of children had the following common features: 94.5% (95%CI, 67.9–74.1) had prostration, 71% (95%CI, 67.9–74.1) vomited during admission, 62% (95%CI, 52.3–65.9) had a Blantyre coma score of 3 or 4, and 57% (95%CI, 51.4–67.5) had convulsions during admission. Hypoglycemia was present in 17% (95%CI, 14.0–19.8) and jaundice was present in 7% (95%CI, 5.8–9.4) of these children. The mortality rate in this group was 4.3% (95%CI, 2.9–5.6).

### Clinical Variables Associated with Death from Severe Malaria

We calculated the prevalence and mortality rates for each of the WHO SM criteria (Table 2). Impaired consciousness (BCS <5), repeated convulsions, coma (BCS <3), and respiratory distress were common presenting signs, each associated with increased risk of death (P<0.001, see Table 2 and Table 3). Severe anemia was present in 24% of cases and was associated with a mortality of 8.6%; however, most of the children who died with severe anemia also had concurrent respiratory distress and/or coma. Hyperparasitemia, hypotensive shock and renal failure were uncommon features of SM in this study (<5% prevalence) that were each associated with increased risk of death. Patients with renal failure had the greatest mortality (52.9%, RR 5.1 [95% CI, 3.2–8.0]). Hyperpyrexia was present in 1.9% of patients and was not significantly associated with death (RR 1.3, [95% CI, 0.7–2.3]).

**Figure 1:**
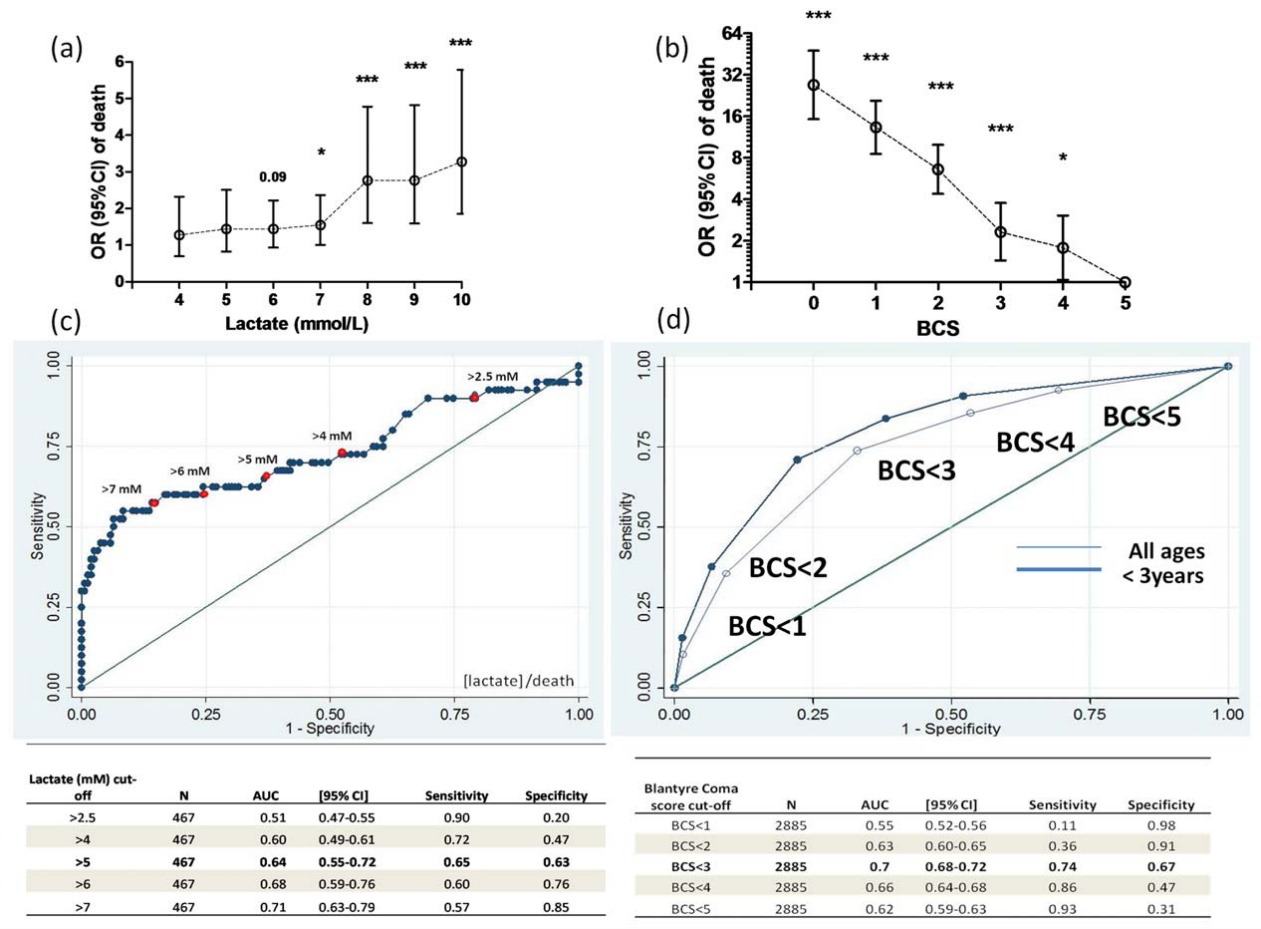
Clinical features associated with death in children with SM. Odds of death and blood lactate concentration in children with SM. Data show the odds ratio (95%CI) of death in relation to increasing concentrations of blood lactate in 467 children with SM (a). Odds of death and Blantyre coma score. OR and P values are relative to BCS = 5 (b). Specificity and sensitivity of different blood lactate concentration cut-off values (c) and coma scores measured by BCS (d) to predict death. AUC =  area under the curve,* P<0.05, ***P<0.001.

Lactate was measured in 467 children with SM. 87% of children had a lactate concentration above the normal range (>2.5 mmol/L). Of these 467 children, 55% had a lactate concentration above 5 mmol/L; the mortality in this group was 15.3%. Hyperlactatemia was associated with increased risk of death (OR 3.19, [95%CI, 1.54–6.60]) among children who did not receive blood transfusion. There was no association between hyperlactatemia and increased risk of death among children treated with blood transfusion. A lactate concentration greater than 5 mmol/L had a sensitivity of 65% and a specificity of 63% to predict death from SM. The area under the ROC curve was 0.64 (95%CI, 0.56–0.70) (Figure 1).

### Age Distribution and Mortality among Children with Severe Malaria

**Figure 2:**
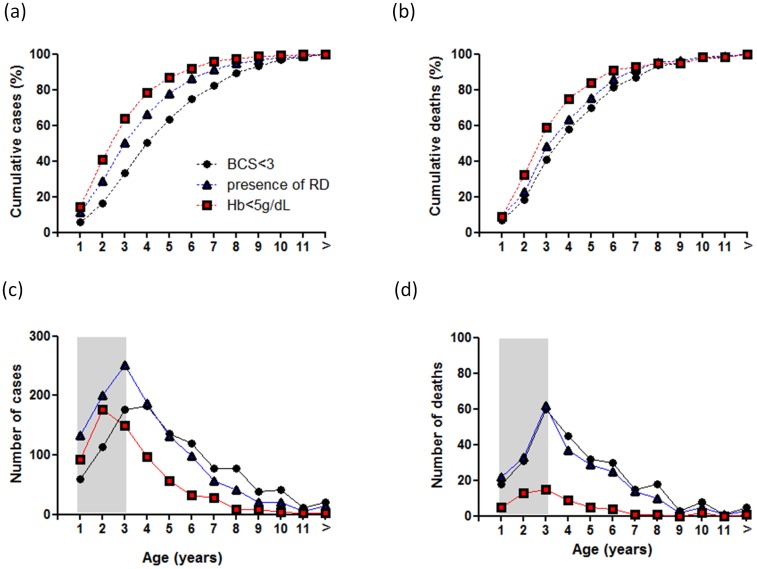
Age distribution of severe malaria syndromes and mortality in Gambian children from 1997 to 2009. Data show the cumulative percentage of children with severe malaria (a), the cumulative percentage of death per age group (b), and absolute number of cases of these SM syndromes (c) and death per age group (d). Age groups indicate year of life: age group 1 includes children up to 12 months of age.

The age distribution and the relationship between age and mortality were different within each of the three major severe malaria syndromes (Figure 2). Children presenting with SM and coma were older (48 months, IQR [34–73]) than those presenting with respiratory distress (36 months, IQR [24–60]) or with severe anemia (30 months, IQR [19–48]). Seventy percent of children with SM (N = 2,045) were younger than 5 years of age. Within the first 5 years of life, we observed 63% of cases of SM with coma (BCS of 2 or less), 78% of respiratory distress and 86% of cases with severe anemia. The number of deaths and the number of cases followed the same trend in children with respiratory distress or severe anaemia. The highest number of deaths observed in children with coma occurred 12 months earlier than the peak number of cases. However, the median age of children with coma who died (47 months, IQR [30–69]) was not significantly different from that of children who survived (51 months, IQR [35.5–80]).

### Blantyre Coma Score is a Quantitative Predictor of Death and Neurological Sequelae

The Blantyre coma score (BCS) was inversely associated with death (Figure 1). Patients with a normal verbal and motor response to a painful stimulus (BCS 5) had a mortality of 3.6% (95% CI, 2.3–4.9%). As the coma score decreased from 4 to 0, the mortality rate increased with each step: 6% (95%CI, 4.0–8.6), 8% (95%CI, 5.8–10.3), 20% (95%CI, 17.1–22.8), 33% (95%CI, 28.1–39.0), and 51% (95%CI, 39.6–61.5). In the logistic regression analysis, the odds of death associated with BCS was 1.99 (95% C.I. 1.82–2.18) for each unit decrease in coma score, a finding that withstood adjustment for respiratory distress, hypoglycemia, age and transfusion (OR  = 1.78, [95%CI, 1.59–1.99]).

To assess the ability of the BCS to predict death, we explored the sensitivity and specificity of different BCS thresholds (Figure 1). The highest sensitivity was achieved using a BCS cut-off value of 4 or less (93%) but the specificity was low (31%). A BCS of 3 or less increased the sensitivity (86%) but lowered the specificity (47%) of this clinical feature to predict death. A BCS of 2 or less had a sensitivity of 74% and specificity of 67% to predict death and provided the greatest area under the ROC curve (0.70, [95%CI, 0.68–0.72]). Among children under 3 years of age, the specificity of this BCS threshold to identify children at risk of dying improved without loss of sensitivity (sensitivity 74%, specificity 77%, AUC 0.74, [95% CI 0.71–0.76]).

Among the children with SM who survived and were neurologically assessed (N = 2,275), 74 (10.4%, [95%CI, 8.1–12.6]) had neurological sequelae at the time of hospital discharge. Most of these patients (49/74) presented with a BCS of 2 or less, and almost all of them (71/74) had a BCS of 4 or less at the time of admission. Blantyre coma score and witnessed convulsions were each independently associated with the development of neurological sequelae (one unit decrease in BCS: OR 1.9 [95%CI, 1.5–2.5]; witnessed convulsion: OR 4.1 [95%CI, 2.2–7.5]). Witnessed convulsions alone had a sensitivity of 78% and a specificity of 64% to predict development of neurological sequelae (area under the ROC curve 0.71 [95%CI, 0.66–0.76)]) whereas the sensitivity and specificity of BCS 2 or less were 74% and 69%, respectively (AUC 0.72 95% CI, 0.67–0.76) (Figure S3).

## Discussion

Despite recent trends showing a decline in the number of children admitted to Gambian hospitals with malaria, [12] severe malaria remains the principal cause for hospital admission of young children. We analyzed clinical data from 2,901 Gambian children admitted to hospital for severe malaria, and we identified features with the greatest sensitivity and specificity to predict in-hospital mortality and development of neurological sequelae. We found the Blantyre coma score to be a quantitative predictor of the risk of death. A Blantyre coma score threshold of 2 or less was the most sensitive and specific predictor death, especially among children younger than 3 years of age. A Blantyre coma score threshold of 2 or less was also the best predictor of the development of neurological sequelae.

The WHO severe malaria case definition captures a highly heterogeneous population with a wide range of mortalities. [5] This clinical definition has a high sensitivity to identify those children at greatest risk of dying to ensure prompt clinical management. However, a sensitive case definition that lacks specificity may overwhelm a high-acuity pediatric ward with admissions that do not require such a high level of care. Similarly, a poorly specific definition may compromise the interpretation of clinical studies or randomized controlled trials where patient enrolment is based on a broad clinical definition. This case definition may be further compromised by co-morbidities.[13]–[16] Previous efforts have applied *P. falciparum* parasite density cut-off values to increase the specificity of the case definition of severe malaria. [17] In this study, we have indentified prognostic factors of severe malaria in Gambian children and assessed their diagnostic performance based on both sensitivity and specificity.

The prevalence of features associated with death in SM was directly correlated with their sensitivity and inversely correlated with their specificity to predict outcome. In this study, the proportion of clinical features independently associated with death ranged from 0.6% for renal failure to 72% for impaired consciousness. Indeed, impaired consciousness was present in most cases that died and thus had a good sensitivity to predict death (92%). However, this feature was also present in 70% of those children who survived, which accounts for its poor specificity (30%). Impaired consciousness includes a heterogeneous population of children with clinical presentations ranging from prostration to deep coma. The Blantyre coma score adequately captures this heterogeneity in an ordinal variable that reflects different degrees of consciousness. We therefore assessed the value of the Blantyre coma score to predict mortality and found the score to be a quantitative predictor of death. Indeed, the current definition of cerebral malaria, a BCS of 2 or less, predicted mortality with good sensitivity and specificity. More importantly, the sensitivity and specificity of this clinical feature increased to 71% and 77%, respectively, in children less than 3 years of age. Similarly, a BCS of 2 or less was a good predictor of subsequent development of gross neurological sequelae.

High lactate concentration commonly underlies metabolic acidosis associated with respiratory distress. [2], [18] We found that high lactate concentration (>2.5 mM) predicted death with high sensitivity and that increasing cut-off values of plasma lactate increased the specificity of lactate to predict death. In the initial analysis we did not find an association of hyperlactatemia and death. This unexpected finding was explained by the large proportion of children with severe malaria and respiratory distress that received blood transfusions and the positive effect of blood transfusion in the outcome of these patients. Indeed, we found hyperlactatemia to be associated with a 3-fold increase in mortality in children who did not receive blood transfusions. However, in these children the prognostic performance of BCS of 2 or less was higher than that of hyperlactatemia, with an associated 5-fold increase in mortality.

A coma score of 2 or less was a better predictor of death in younger children with severe malaria. This finding was expected as the clinical presentation of severe malaria and syndrome-specific mortality rates vary in different epidemiological settings with different transmission intensities. [6], [19] These data show that the peak prevalence of cerebral malaria occurs a year later than severe malarial anemia and respiratory distress as previously described for this and other settings. [6], [20] Also, unlike other severe malaria syndromes, older Gambian children continued to acquire cerebral malaria. However, the number of deaths paralleled those of respiratory distress. Indeed, we found the highest case fatality rates in those children who presented with both cerebral malaria and respiratory distress. After adjusting for age and transfusion, the mortality of these syndromes combined was nearly nine times higher than the other severe malaria syndromes and 3 times higher than that of cerebral malaria only (see Figure S2). The high mortality rates associated with severe malaria described in this study have been reported previously in The Gambia and other sites in Sub-Saharan Africa. [7], [21] It is possible that at times when multiple children present simultaneously to the RVTH, our study personnel prioritized enrollment of the most severely ill patients, thus enriching the study with patients in the severe spectrum of malarial illness. However, our enrollments were representative of all malaria admissions to RVTH in 2003 when data was available for comparison. [7] The prevalence of coma, severe anemia and respiratory distress at the time of admission in our study was also similar to the prevalence reported in a recent African hospital-based multi-center drug trial (AQUAMAT). [10], [22].

This study is a large scale validation of the Blantyre Coma Score and underscores the prognostic importance of impaired consciousness in children with severe malaria. In Gambian children, a BCS of 2 or less outperforms any other clinical feature to predict death.

## Supporting Information

Figure S1 **Number of patients enrolled in the study and proportions of death in patients admitted with severe malaria from 1997 to 2009.** Non-SMS (non-severe malaria syndrome).
(TIF)

Figure S2 **Prevalence and mortality of overlapping severe malaria syndromes.** Diagram shows number of cases and number of deaths per syndrome in Gambian children with severe malaria. Multiple logistic regression analysis show the OR (95%CI) of death for unique and overlapping severe malaria syndromes.
(TIF)

Figure S3 **Predictors of neurological sequelae at discharge from hospital in Gambian children with SM.** Data show the sensitivity and specificity of different BCS cut-off values and the presence of convulsions during admission (CONV).
(TIF)
