# Supplementary figures and images for: Clinical Features of Severe Malaria Associated with Death: A 13-Year Observational Study in The Gambia
Source: PLoS One. 2012 Sep 28;7(9):e45645. doi: 10.1371/journal.pone.0045645 (PMC3460946; doi:10.1371/journal.pone.0045645)

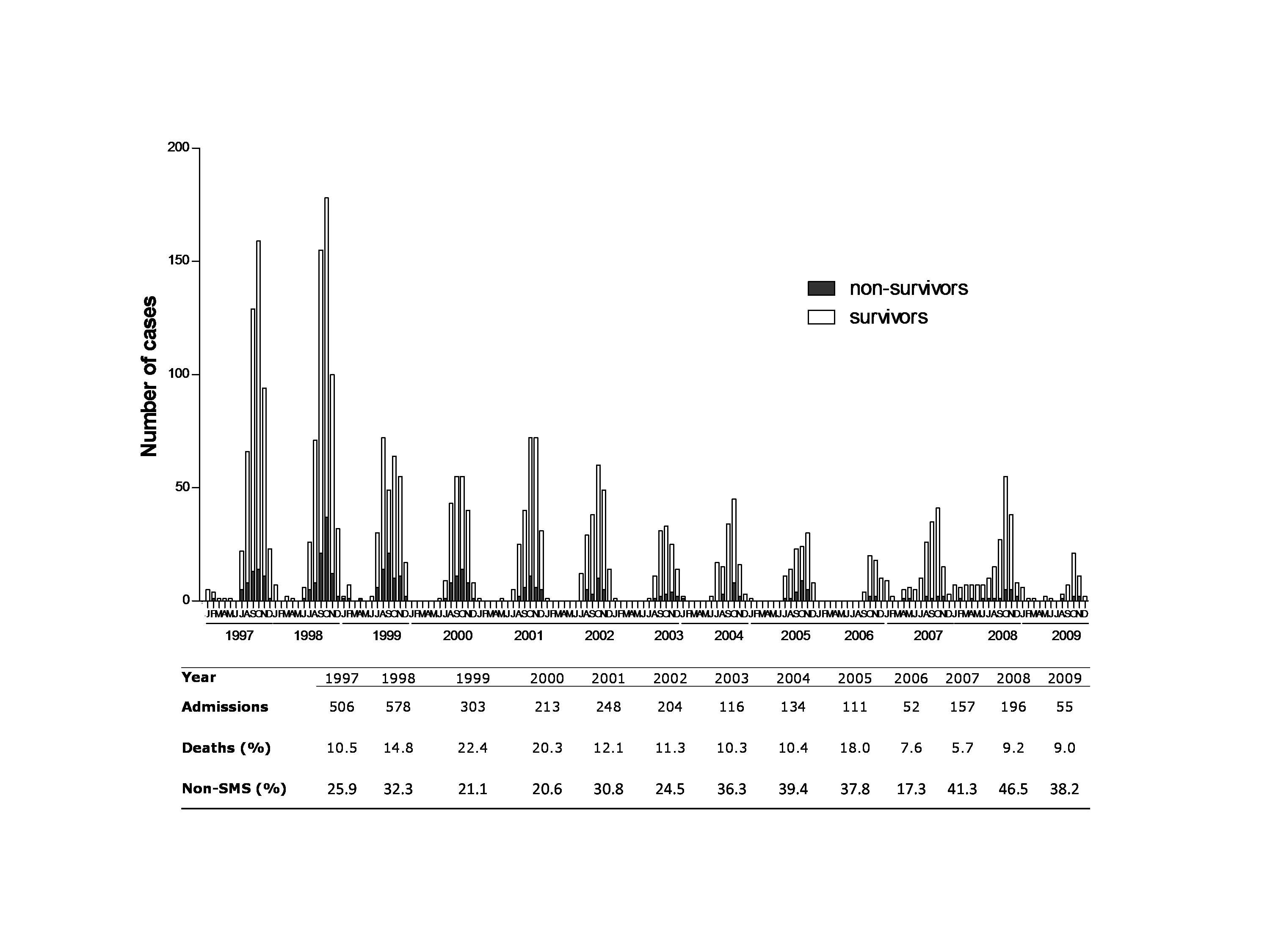

Supplement: Figure S1 — Number of patients enrolled in the study and proportions of death in patients admitted with severe malaria from 1997 to 2009. Non-SMS (non-severe malaria syndrome). (TIF) [file pone.0045645.s001.tif]

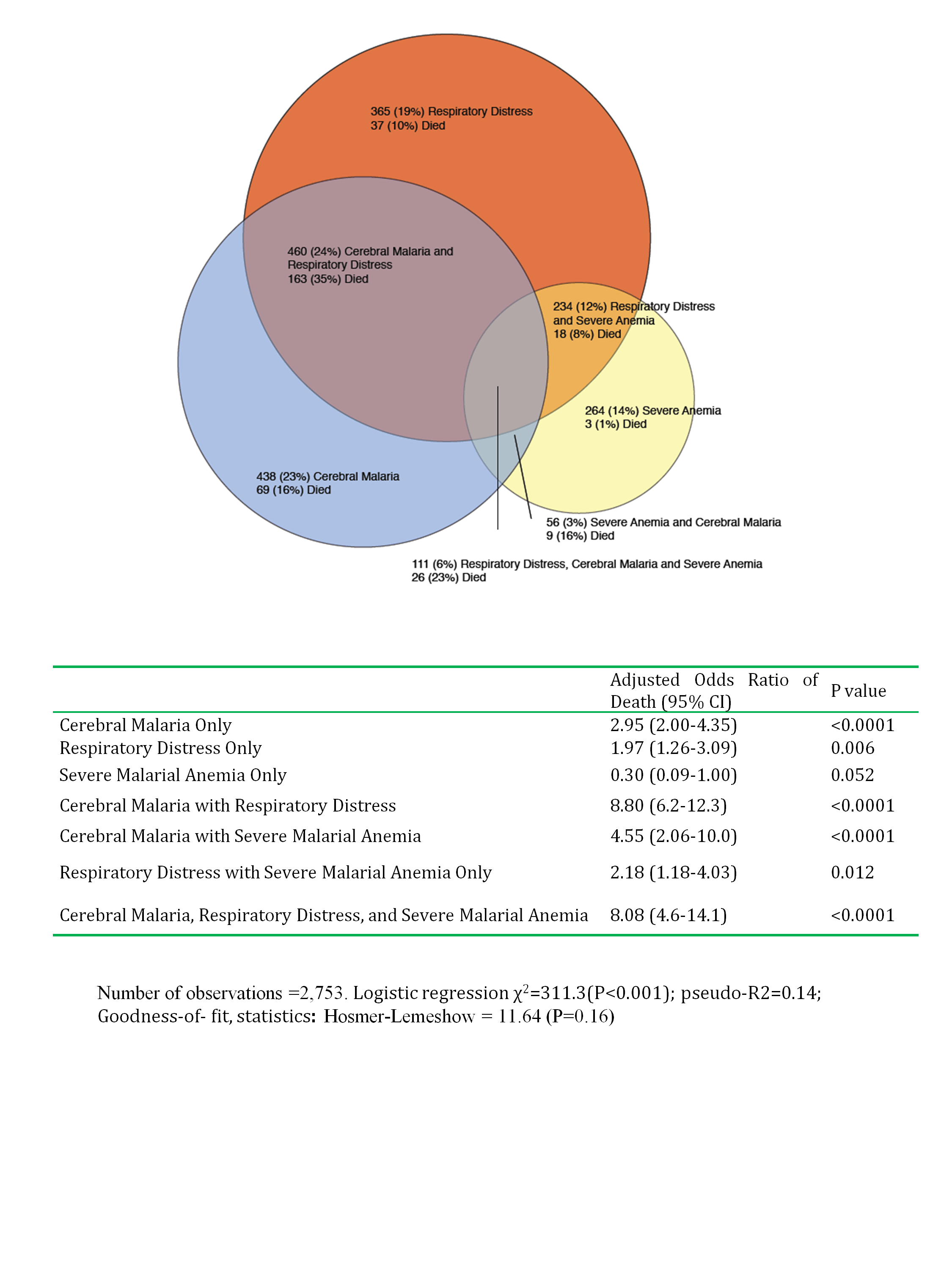

Supplement: Figure S2 — Prevalence and mortality of overlapping severe malaria syndromes. Diagram shows number of cases and number of deaths per syndrome in Gambian children with severe malaria. Multiple logistic regression analysis show the OR (95%CI) of death for unique and overlapping severe malaria syndromes. (TIF) [file pone.0045645.s002.tif]

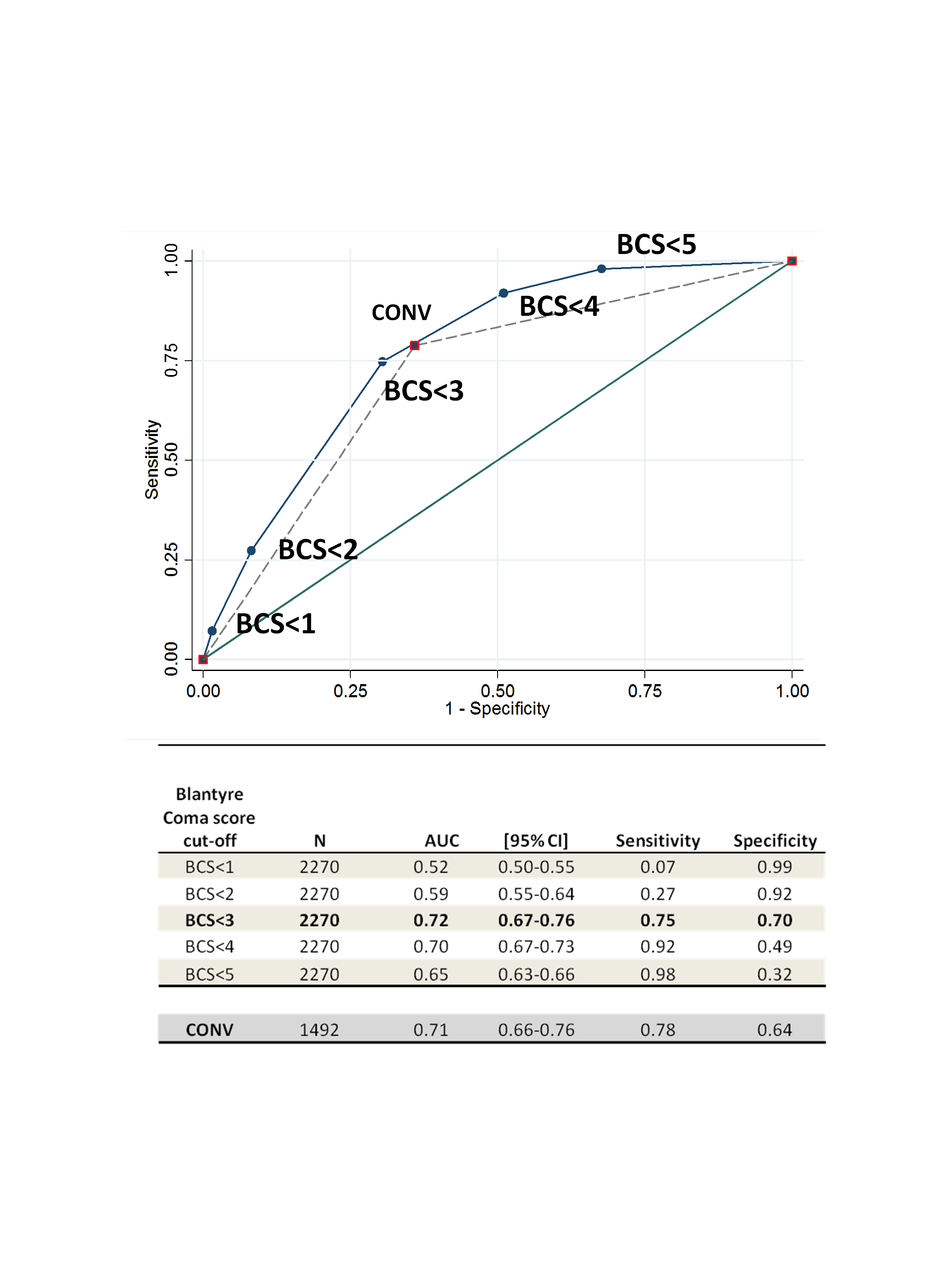

Supplement: Figure S3 — Predictors of neurological sequelae at discharge from hospital in Gambian children with SM. Data show the sensitivity and specificity of different BCS cut-off values and the presence of convulsions during admission (CONV). (TIF) [file pone.0045645.s003.tif]
